# Electrochemical Fingerprint Biosensor for Natural Indigo Dye Yielding Plants Analysis

**DOI:** 10.3390/bios11050155

**Published:** 2021-05-14

**Authors:** Boyuan Fan, Qiong Wang, Weihong Wu, Qinwei Zhou, Dongling Li, Zenglai Xu, Li Fu, Jiangwei Zhu, Hassan Karimi-Maleh, Cheng-Te Lin

**Affiliations:** 1 Key Laboratory of Novel Materials for Sensor of Zhejiang Province, College of Materials and Environmental Engineering, Hangzhou Dianzi University, Hangzhou 310018, China; 191200023@hdu.edu.cn (B.F.); whwu@hdu.edu.cn (W.W.); zhouqw@hdu.edu.cn (Q.Z.); 2 Institute of Botany, Jiangsu Province & Chinese Academy of Sciences (Nanjing Botanical Garden Mem. Sun Yat-Sen), Nanjing 210014, China; wangqiong@cnbg.net (Q.W.); lidongling@cnbg.net (D.L.); xuzenglai@cnbg.net (Z.X.); 3 The Jiangsu Provincial Platform for Conservation and Utilization of Agricultural Germplasm, Nanjing 210014, China; 4 Co-Innovation Center for Sustainable Forestry in Southern China, Nanjing Forestry University, Nanjing 210037, China; jwzhu@njfu.edu.cn; 5 School of Resources and Environment, University of Electronic Science and Technology of China, Xiyuan Ave, Chengdu 611731, China; hassan@uestc.edu.cn; 6 Department of Chemical Engineering, Quchan University of Technology, Quchan 9477177870, Iran; 7 Department of Chemical Sciences, Doornfontein Campus, University of Johannesburg, P.O. Box 17011, Johannesburg 2028, South Africa; 8 Key Laboratory of Marine Materials and Related Technologies, Zhejiang Key Laboratory of Marine Materials and Protective Technologies, Ningbo Institute of Materials Technology and Engineering (NIMTE), Chinese Academy of Sciences, Ningbo 315201, China; linzhengde@nimte.ac.cn

**Keywords:** electroanalysis, indigo dyes, fast identification, fingerprints, differential pulse voltammetry

## Abstract

Indigo is a plant dye that has been used as an important dye by various ancient civilizations throughout history. Today, due to environmental and health concerns, plant indigo is re-entering the market. *Strobilanthes* *cusia* (Nees) Kuntze is the most widely used species in China for indigo preparation. However, other species under *Strobilanthes* have a similar feature. In this work, 12 *Strobilanthes* spp. were analyzed using electrochemical fingerprinting technology. Depending on their electrochemically active molecules, they can be quickly identified by fingerprinting. In addition, the fingerprint obtained under different conditions can be used to produce scattered patter and heatmap. These patterns make plant identification more convenient. Since the electrochemically active components in plants reflect the differences at the gene level to some extent, the obtained electrochemical fingerprints are further used for the discussion of phylogenetics.

## 1. Introduction

Plant indigo was the most widely used and important dye in the world until the invention of synthetic organic dye aniline violet in 1856. India is widely believed to be the oldest centre of indigo dyeing, and has been Europe’s most important importer of the dye since Greco-Roman times [1,2,3,4]. Tombs in Egypt have unearthed linen fabrics from around 2400 BC, some of them with delicate indigo lace. In ancient Israel and Palestine, indigo was mixed with green and black dyes for dyeing. Historians in the Egyptian town of Forstadt, south of Cairo, where caravans used to stop during the Middle Ages, have unearthed large quantities of chintz fragments from India [5,6,7,8]. Some of them were printed with plant indigo. China, along with Egypt, Peru and India, is the ancient country that applies indigo in the world. Due to the humid climate in Asia, natural fibers are easily damaged. Therefore, ancient fabrics are difficult to preserve in historical relics. However, there are still many discoveries that have been made through archaeological work. Indigo was used to dye silk fabrics unearthed from the Western Han Dynasty tomb in Mawangdui, Changsha. Silk and cotton printing and dyeing products of the Tang Dynasty are preserved in Shosakura in Xinjiang and Japan, including batik cotton fabric with blue background and white flower [9,10,11,12].

Indigo is still widely used in traditional textiles, even though other natural plant dyes are rarely used. *Strobilanthes cusia* (Nees) Kuntze is the most widely used species in China for indigo preparation [13,14]. *Strobilanthes* spp. is the second largest genus of the family Acanthaceae distributed in tropical and subtropical regions of Asia. Estimates of the number of species in *Strobilanthes* Blume range from 250 to 450. Many of these species are also used to make indigo. Since many of these species are morphologically similar, identifying plants is not an easy task among non-botanists. With the development of digital image processing and recognition technology, digital images of plants are often used for species identification [15,16]. This approach can be used effectively in species with large morphological differences. However, it is unable to distinguish between species with very similar morphology, and it is especially difficult to do so in the same genus [17]. In this case, spectral analysis and chemical signal analysis are able to overcome this difficulty. For example, Fourier transform infrared (FTIR) spectroscopy allows for the classification of plants based on their different phytochemical compositions [18,19]. However, FTIR spectra mainly reflect certain groups or bonds in the molecule, such as methyl, methylene, carbonyl, cyano, hydroxyl and amine groups. The differences in the spectra in fact reflect differences in the composition of the functional groups and, therefore, do not fully respond to the differences in chemical composition [20]. Conversely, electrochemical fingerprinting can reflect the variability in electrochemically active molecules in the detection system. Previous studies have shown that electrochemical fingerprinting can be successfully applied in plant identification and phylogenetic studies [21,22]. This technology has the potential to be developed for the identification of different commercial plants and the monitoring of corresponding products.

In this study, we selected 12 species from *Strobilanthes* and 2 exotaxa for analysis. Electrochemical taxonomy is a new recently developed technology and is used as an alternative method for plant phylogenetics analysis [23,24,25,26,27,28]. The electrochemical fingerprints of these species were recorded under different conditions. The patterns of these species were generated for identification. Then, the phylogenetic position of these species was studied.

## 2. Materials and Methods

*Strobilanthes hossei, Strobilanthes japonica, Strobilanthes dimorphotricha, Strobilanthes cusia, Hemigraphis cumingiana, Strobilanthes oliganthus, Strobilanthes hamiltoniana, Strobilanthes austrosinensis, Strobilanthes henryi, Strobilanthes tonkinensis, Strobilanthes schomburgkii, Strobilanthes dyeriana, Strobilanthes hamiltoniana, Strobilanthes biocullata and Peristrophe japonica* were supplied by Nanjing Botanic Garden. All chemicals were analytical grade and used without purification. All electrochemical fingerprint recordings were conducted using a CHI760 electrochemical workstation. A commercial glassy carbon electrode (GCE), an Ag/AgCl electrode and a Pt electrode were used as the working electrode, reference electrode and counter electrode, respectively.

Ethanol and water were used as solvents for plant leaf extraction. A small amount of leaf (0.01 g) was carefully mixed with 2 mL of solvent. Then, the slurry was sonicated for 5 min for extraction. Then, 2 μL of plant tissue dispersion was drop coated on the working electrode surface and dried naturally. In this study, the electrochemical fingerprinting of two conditions was recorded. The samples extracted with water were recorded under PBS. Samples extracted with ethanol were recorded under ABS. The voltammetric profile (fingerprints) of plant leaf were recorded using differential pulse voltammetry (DPV) in the range −0.1 to 1.5 V in either PBS (pH 7.0, 0.1 M) or ABS (pH 4.5, 0.1 M). Except for the reproducibility test, the fingerprints of herbal tissue were recorded repeated three times in each condition.

All raw data were first treated with a normalization process, where the ratios between the current and the maximum peak current were obtained at different potentials (Scampicchio et al., 2005). The normalized voltammetric data have been used for pattern generation. PCA analysis and cluster analysis were carried out using Origin 2021. The ward linkage method was applied during the cluster analysis.

## 3. Results and Discussion

**Figure 1:**
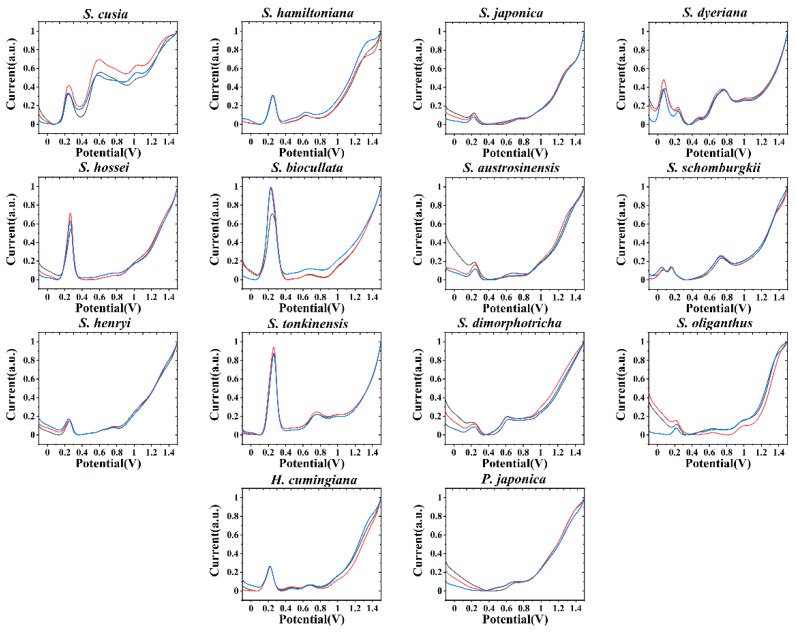
Electrochemical fingerprint of *S. hossei, S. japonica, S. dimorphotricha, S. cusia, S. biocullata, S. oliganthus, S. hamiltoniana, S. austrosinensis, S. henryi, S. tonkinensis, S. schomburgkii, S. dyeriana, S. hamiltoniana, H. cumingiana* and *P. japonica* recorded after water extraction in PBS.

Figure 1 shows the voltammetric profiles of *S. hossei, S. japonica, S. dimorphotricha, S. cusia, S. biocullata, S. oliganthus, S. hamiltoniana, S. austrosinensis, S. henryi, S. tonkinensis, S. schomburgkii, S. dyeriana, S. hamiltoniana, H. cumingiana* and *P. japonica* recorded after water extraction in PBS. It can be seen that each DPV curve has electrochemical oxidation signal, which represents that each species contains electrochemical active molecules [29,30,31]. According to phytochemical studies [32,33,34], these electrochemically active molecules are phenolic acids, alkaloids, pigments, flavonols and procyanidins. Although we have no way to distinguish each of these molecules in the electrochemical fingerprinting, since many of them have similar chemical structures and are able to oxidize at similar potentials. However, the height and area of these electrochemical oxidation peaks have a positive correlation with the type and number of oxidized molecules. Therefore, comparing the differences in the electrochemical fingerprints of plants can distinguish the differences in electrochemically active molecules in plants. No curves of two samples showed exact same profile, representing the difference of the molecules involved in the electrochemical reaction in each species. This difference is due to differences in the composition and number of electrochemical molecules in different species. The differences also reflect differences at the genetic level [35,36,37]. Although the composition of a plant is influenced by its environment, is primarily determined by its genes. Three samples have been tested for each species and found to be well reproducible. Therefore, differences in these DPV profiles can be used to identify different species based on the peak locations and peak intensity. However, we see some similarities in the fingerprints of some species, such as *S. hossei* and *S. biocullata*. These two species are not only very similar in leaf morphology, but their flowers are also very similar in morphology and color [38,39].

**Figure 2:**
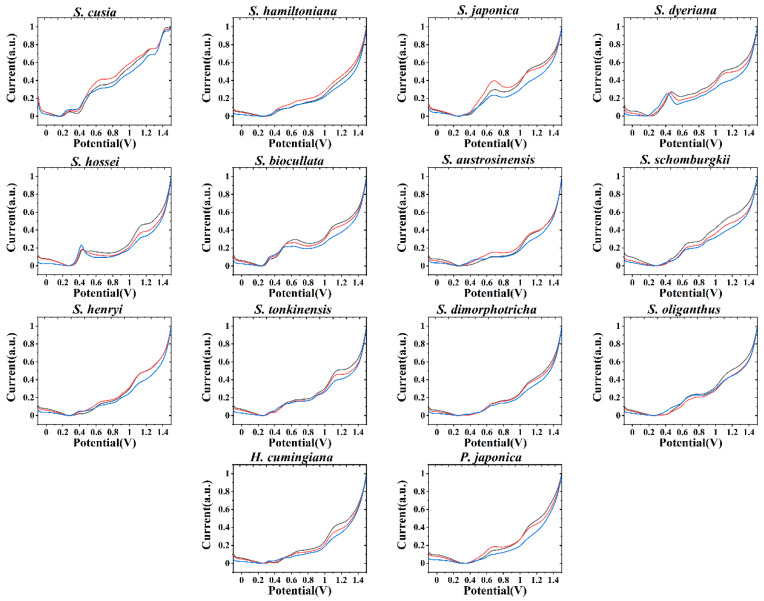
Electrochemical fingerprint of *S. hossei, S. japonica, S. dimorphotricha, S. cusia, S. biocullata, S. oliganthus, S. hamiltoniana, S. austrosinensis, S. henryi, S. tonkinensis, S. schomburgkii, S. dyeriana, S. hamiltoniana, H. cumingiana* and *P. japonica* recorded after ethanol extraction in ABS.

In order to increase the accuracy of recognition, ethanol was used to extract plant tissues, and fingerprint was collected in ABS. As shown in Figure 2, each species also exhibits electrochemical oxidation behavior under these conditions. Studying Figure 1, it can be seen that the electrochemical oxidation behavior of each plant is not consistent. This is for two reasons. The first is that the species produce different electrochemically active molecules in the extraction of different solvents [40]. Another reason is that in, different pH and buffer solution environments, the oxidation potential of the molecules involved in electrochemical oxidation is not the same [41]. This allows the two species with similar electrochemical behavior, such as *S. hossei* and *S. biocullata*, to exhibit different behaviors here. Therefore, although the two species may be morphologically very similar, their electrochemically active molecules will remain somewhat different in type and amount. Combined with electrochemical fingerprinting, taken under different conditions, this difference can be amplified and provide the opportunity to perform identification.

**Figure 3:**
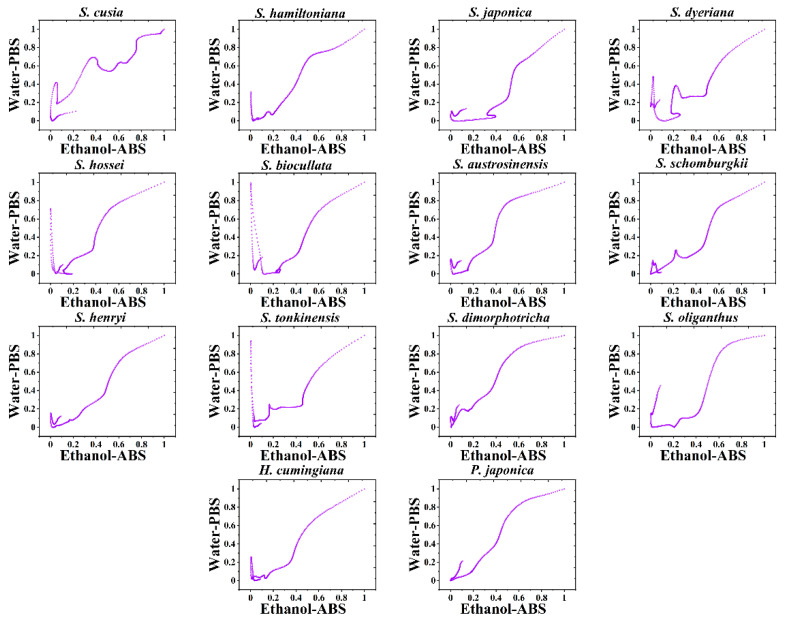
Scatter patterns of *S. hossei, S. japonica, S. dimorphotricha, S. cusia, S. biocullata, S. oliganthus, S. hamiltoniana, S. austrosinensis, S. henryi, S. tonkinensis, S. schomburgkii, S. dyeriana, S. hamiltoniana, H. cumingiana* and *P. japonic*a.

However, it is difficult to directly identify species using DPV profiles, especially when there are many samples. Thus, we combined the data from the two figures to produce a scatter plot pattern for each species. Because the potential information in the two sets of data is exactly the same, we delete their weights. In this case, the scatter plot’s *X*-and *Y*-axis data are given equal weight, and we can combine the species’ electrochemical fingerprints collected in both conditions in a single pattern. Figure 3 shows the scatter patterns of *S. hossei, S. japonica, S. dimorphotricha, S. cusia, S. biocullata, S. oliganthus, S. hamiltoniana, S. austrosinensis, S. henryi, S. tonkinensis, S. schomburgkii, S. dyeriana, S. hamiltoniana, H. cumingiana* and *P. japonica*. As can be seen from the figure, each species has its own unique pattern. By dividing the whole area into several quadrants, and then counting the data points in different quadrants, the unknown sample can be compared with the database. Multivariate variance analysis shows that there is no significant difference between patterns of the same species. However, there are significant differences between the scatter patterns of any two species. Therefore, the scatter pattern is a better recognition pattern than the DPV profile. We conducted the investigation of the reproducibility of scatter pattern. Although all patterns of one species showed a similar shape, a small difference can be observed when overlapping them together. These variations are inevitable for fingerprinting the chemical and metabolic profile of a biological sample. However, the slight differences between individual recordings from the same species cannot affect the identification results. Based on the significant pattern difference between the species, the unknown sample can be compared with the database for identification.

**Figure 4:**
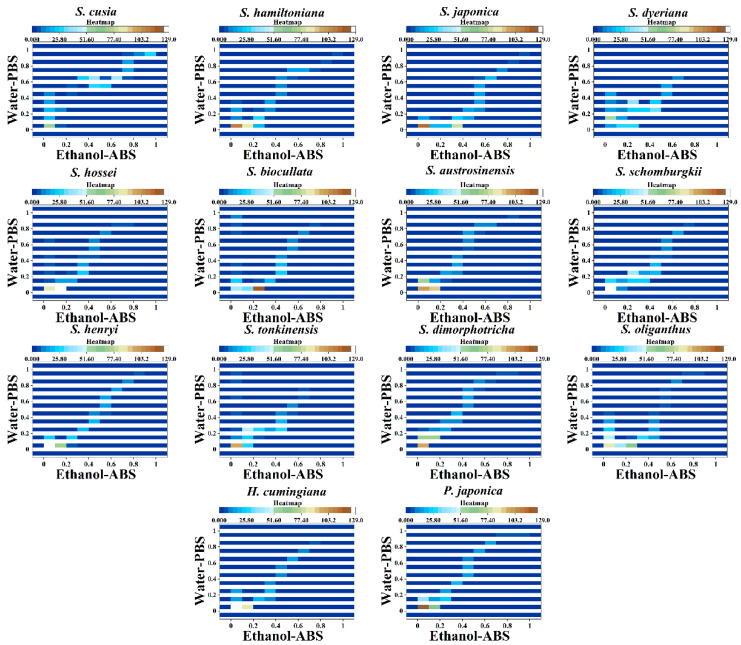
Heatmap of *S. hossei, S. japonica, S. dimorphotricha, S. cusia, S. biocullata, S. oliganthus, S. hamiltoniana, S. austrosinensis, S. henryi, S. tonkinensis, S. schomburgkii, S. dyeriana, S. hamiltoniana, H. cumingiana* and *P. japonica*.

We further propose a more intuitive pattern recognition method. In this model, two sets of fingerprints collected in different environments can be used to make a heatmap of the species. As shown in Figure 4, we not only combined the two groups of data, but also displayed a pattern similar to a scatter pattern. In addition, we put a value on the density of the data. The more data points in an area, the darker hot spots will appear. In this pattern recognition mode, we no longer need logarithmic data points for statistics, but only need to locate the range of the hot area. Species can be identified if the hot zones of a species are in a composite database of unknown samples.

Principal component analysis (PCA) is a common statistical technique used to analyze differences between data groups and between data groups. In this work, we performed a PCA analysis of the homogenized current values collected in both environments for each species.

**Figure 5:**
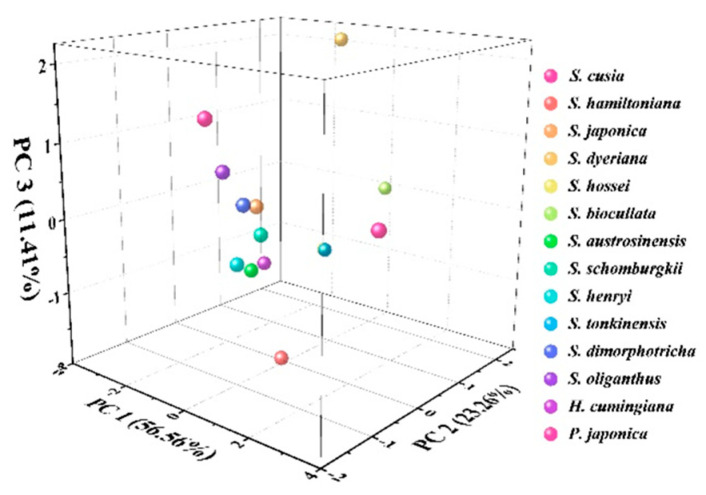
PCA analysis of *S. hossei, S. japonica, S. dimorphotricha, S. cusia, S. biocullata, S. oliganthus, S. hamiltoniana, S. austrosinensis, S. henryi, S. tonkinensis, S. schomburgkii, S. dyeriana, S. hamiltoniana, H. cumingiana* and *P. japonica*.

As shown in Figure 5, *S. Japonica*, *S. Dimorphotricha*, and *S. Schomburgkii* were grouped together. Meanwhile, *S. austrosinensis*, *S. oliganthus*, and *H. cumingiana* were grouped into one cluster. The proximity of their data is due to the similarity of electrochemically active molecules in their tissues. This also reflects their genetic similarity.

**Figure 6:**
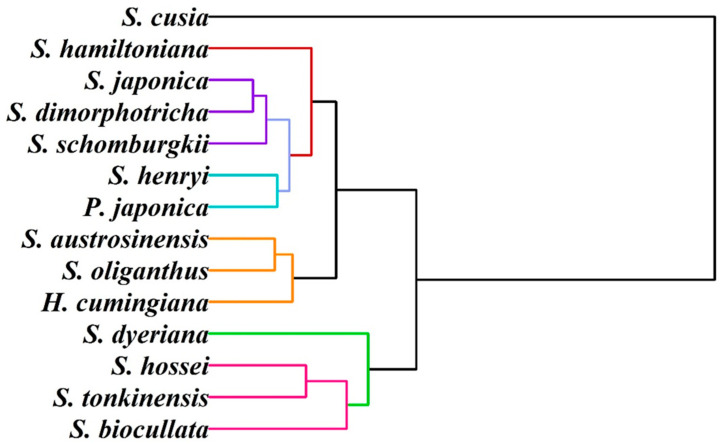
Dendrogram of *S. hossei, S. japonica, S. dimorphotricha, S. cusia, S. biocullata, S. oliganthus, S. hamiltoniana, S. austrosinensis, S. henryi, S. tonkinensis, S. schomburgkii, S. dyeriana, S. hamiltoniana, H. cumingiana* and *P. japonica* based on electrochemical fingerprints.

We further attempted to study the infrageneric relationships of these species, and hierarchical clustering analysis was carried out using electrochemical profiles. As shown in Figure 6, the first group consisted of the species *S. dyeriana, S. hossei, S. tonkinensis* and *S. biocullata*. The second group contains two clades. The clade included *S. austrosinensis, S. oliganthus* and *H. cumingiana*. Another clade included *S. hamiltoniana, S. japonica, S. dimorphotricha, S. schomburgkii, S. henryi* and *P. japonica*. One outlier can be seen in *S. cusia*. This result is not entirely consistent with the results of other taxonomic techniques. This may be due to the confusing taxonomic results of the genus *Strobilanthes*. For example, Bremekamp divided *Strobilanthes* and its allies into over 54 genera arranged in 27 informal groups [42]. Terao recognized a broadly circumscribed *Strobilanthes* comprising all species of Strobilanthinae [43]. The results of recent molecular studies, statistical analysis and pollen and gross morphology showed that these results are problematic [44,45,46,47]. Our results provide a new explanation.

## 4. Conclusions

In this work, we provide an electrochemical method for potential identifying species of the dye plant indigo by using the fingerprints of electrochemically active molecules in plant tissues. Two different conditions were combined using solvents and buffer solutions for the recording of electrochemical fingerprints. The same species exhibit different fingerprint profiles under different conditions because different electrochemically active molecules were extracted and were involved in electrochemical oxidation under different pH conditions. The fingerprint profiles of some species showed similarity under one condition, but went very differently under another condition. Therefore, combining two sets of fingerprint profiles can be used to make a scatter pattern and heatmap for the identification of species. In these two pattern modes, the species were easier to identify than the DPV curves directly. The electrochemical fingerprinting presents information that can be linked to their genetic level. The dendrogram indicated that the 14 species were divided into three main clades. An outlier of *S. cusia* was observed.

## Data Availability

Data sharing not applicable.
